# Effects of Curculigoside on Memory Impairment and Bone Loss via Anti-Oxidative Character in APP/PS1 Mutated Transgenic Mice

**DOI:** 10.1371/journal.pone.0133289

**Published:** 2015-07-17

**Authors:** Lu Zhao, Sha Liu, Yin Wang, Qiaoyan Zhang, Wenjuan Zhao, Zejian Wang, Ming Yin

**Affiliations:** 1 School of Pharmacy, Shanghai Jiaotong University, Shanghai, China; 2 People's Liberation Army (PLA) 455 Hospital, Shanghai, China; 3 Department of Pharmacology, School of Pharmacy, Chengdu Medical College, Sichuan, China; 4 School of Pharmacy, Second Military Medical University, Shanghai, China; University of S. Florida College of Medicine, UNITED STATES

## Abstract

Alzheimer's disease (AD) and osteoporosis are two closely related multifactorial progressively degenerative diseases that predominantly affect aged people. These two diseases share many common risk factors, including old age, being female, smoking, excessive drinking, low estrogen, and vitamin D3 levels. Additionally, oxidative damage and the dysfunction of the antioxidant system play important roles in the pathogenesis of osteoporosis and AD. Aβ not only leads to impaired memory but also plays a crucial role in the demineralization process of bone tissues of older people and women with menopause. Curculigoside can promote calcium deposition and increase the levels of ALP and Runx2 in osteoblasts under oxidative stress via anti-oxidative character. Therefore, we investigated the effects of CUR on the spatial learning and memory by the Morris water maze and brain immunohistochemistry, and bone microstructure and material properties of femurs by micro-computed tomography and mechanical testing in APP/PS1 mutated transgenic mice. Oral administration of CUR can significantly enhance learning performance and ameliorate bone loss in APP/PS1 mutated transgenic mice, and the mechanism may be related to its antioxidant effect. Based on these results, CUR has real potential as a new natural resource for developing medicines or dietary supplements for the prevention and treatment of the two closely linked multifactorial progressive degenerative disorders, AD and osteoporosis.

## Introduction

Osteoporosis is a progressive bone degenerative diseases characterized by decreases in bone mass and bone mineral density (BMD), as well as deteriorates in bone microarchitecture which can lead to an increased risk of fracture [1]. The intimate cooperation of bone cells including osteoblasts, osteoclasts and osteocytes maintains the integrity of bone structure [2]. The traditional understanding of the pathogenesis of osteoporosis is that the cessation of ovarian function at menopause is responsible for the accelerated rate of postmenopausal bone loss [3]. However, previous studies have suggested that increased level of reactive oxygen species (ROS) has more close relationship with the pathogeny of the bone age-related diseases [4]. Increasing clinical studies show that senile dementia patients are more likely to suffer from osteoporosis and have higher risk of hip fracture [5]. AD transgenic mice also have decreased levels of osteoblastogenesis and loss of trabecular bone mass [6]. Abnormal amyloid beta peptide (Aβ) deposition is one of the most important pathological factors of AD. The mRNA and protein expression levels of Aβ42 and the amyloid precursor protein (APP) were elevated remarkably in the bone of osteoporosis patients and ovariectomized rats [7]. The oxidative damage induced by Aβ deposition may be a common pathogenic factor of the two closely related degenerative diseases [8].

*Curculigo orchioides* belongs to the Amaryllidaceae family, which has been considered to one of the important herbal medicine in the traditional Chinese medical system. The major biologically active ingredient present in *Curculigo orchioides*, curculigoside (CUR), has been found to show wide spread pharmacological activities including anti-immunostimulation [9], oxidation resistance [10,11], angiogenesis [12], anti-osteoporosis [13], and neuroprotection [14]. In our previous study, CUR reduced the oxidative damage and induced proliferation and differentiation of osteoblasts under oxidative stress status, as well as inhibited bone resorption via its anti-oxidative character in ovariectomized rats [15,16,17]**。**. In addition, CUR can also improve the learning and memorizing ability of aged rats by decreasing cerebral acetylcholinesterase activity and inhibiting the expression of β-site APP cleaving enzyme 1 in the hippocampus [18]. In this study, we investigated the effect of CUR on Aβ deposition induced memory deficit, bone loss and the potential proximate mechanisms.

## Materials and Methods

### 2.1 Chemicals

CUR (purity. 98%) was isolated from *Curculigo orchioides* [19]. The mouse anti-osteocalcin (OCN) antibody was purchased from Millipore (Bedford, MA). The mouse anti-FOXO1 antibody and β-Amyloid antibody were purchased from Cell Signaling Technology (Beverly, MA), and other antibodies were purchased from Abcam (Cambridge, MA). N-acetyl-L-cysteine (NAC) and all others chemicals were purchased from Sigma.

### 2.2 Animals

APP/PS1 mutated transgenic mice used in the present study were obtained from the Jackson Laboratory (stock no. 004462). Animal studies were approved by the Animal Research Committee of the Shanghai Jiao Tong University, and were carried out under the Guidelines for Animal Experiment of the Shanghai Jiao Tong University (Approval No. SYXK 2012–0017, Shanghai, China). The experimental animals were housed in hygienic plastic cages in a clean well-ventilated room and were given free access to food and water with normal light and dark cycles.

### 2.3. *In vivo* study design

Forty mice (9 month old) utilized in this study were divided into 4 groups with 10 in each: wild type (C57BL/6J), APP/PS1 (C57BL/6J), APP/PS1 (C57BL/6J) treated with NAC (100 mg/kg), and APP/PS1 (C57BL/6J) treated with CUR (100 mg/kg). CUR and NAC were dissolved by 0.5% CMC-Na into liquid suspension respectively, with concentration of 9 mg/ml. CUR and NAC were intragastrically administrated to APP/PS1 mutated transgenic mice for four weeks. Four weeks later, the mice were sacrificed after behavior experiments and blood was collected for the measurement of cytokines. Femurs were collected for micro-computed tomography scanning, mechanical testing, and antioxidant enzymes assay. Brains were collected for immunohistochemistry and antioxidant enzyme assay.

#### 2.3.1 Morris water maze

Spatial learning and memory of mice were assessed by the Morris water maze consisted of a circular pool (1.8 m in diameter) as described [20]. Water in the circular pool was held at 24 ± 0.5°C. The escape platform (9 cm in diameter) was submerged 1.5 cm below the water in the target quadrant (SW 3). Each mouse was trained for 4 trials per day for 5 consecutive days. During the training, the time to find the platform was recorded as latency. On the sixth day, the platform was removed to measure memory retention of the mice in four groups. Each mouse monitored by a video camera had 60 s to investigate the target quadrant. The time spent in the target quadrant was analyzed.

#### 2.3.2 Immunohistochemistry

The right hemispheres of the mice in four groups were fixed in 4% paraformaldehyde, and then placed in 30% sucrose until sinking to the bottom. The hemispheres were cut and every section (10 μm) was stained with β-Amyloid antibody to recognize total Aβ plaques (including several isoforms of beta-amyloid peptide, such as Aβ-40, Aβ-42) as described [21]. The sections were mounted on slides for immunofluorescence detection using an Olympus microscope with DP-70 software.

#### 2.3.3 Micro-Computed Tomography

The left femurs of the mice were fixed in 4% paraformaldehyde, and then placed with gauze in the sample holder and scanned with GE Healthcare Locus SP micro CT (GE Healthcare, USA) using 6 μm resolution, 80 kV, 80 μA, 400 views and 5 hours of exposure time [22]. The explore reconstruction utility software (GE Healthcare,USA) was used for three-dimensional reconstruction and data processing. Calculation methods of bone parameters have been previously described [23]. The BMD (bone mineral density), bone volume fraction (BVF), trabecular thickness, trabecular number, and trabecular spacing of femurs were analyzed to evaluate bone quality of femurs.

#### 2.3.4 Mechanical testing

To assess the effects of CUR treatment on the mechanical properties of femurs, three-point bending testing was performed using a Dynamic Mechanical Analyzer (Shimadzu, Japan) to determine the material properties including elastic load, maximum load, elastic stress, maximum stress and modulus of elasticity of the bones. Right femurs of the mice were stored at −20°C and allowed to equilibrate to room temperature in saline for 30 minutes prior to mechanical testing. The femurs were loaded with a span length of 6 mm at a rate of 0.1 mm/s until the moment of fracture [24]. The load-time curve obtained was converted into a load displacement curve, and the material properties were calculated according to formulas [25].

#### 2.3.5 Antioxidant enzymes assay

Brain hemispheres and femurs were collected and stored at − 80°C for later use [26]. The samples were homogenized in 4 volumes of Tris-HCl buffer, and then centrifuged at 5000×g for 15 min. The supernatant was collected and determined within 2 h. The activities of catalase (CAT), superoxide dismutase (SOD) and the concentration of malondialdehyde (MDA) in brain and femurs were examined using commercial kits according to the manufacturer’s instructions (Jiancheng, Nanjing, China).

#### 2.3.6 ELISA analysis

The left hemispheres of the mice from four groups were homogenized in ice-cold PBS containing 5 M guanidine hydrochloric acid and protease inhibitor mixture (Roche Diagnostics). The femurs extracts were prepared by extracting frozen pulverized bone tissue and suspending in ice-cold PBS containing 5 M guanidine hydrochloric acid and protease inhibitor mixture (Roche Diagnostics). Total protein content in the brain hemispheres and femurs extracts was determined via colorimetric BCA assay in accordance with the manufacturer’s recommendations (Jiancheng, Nanjing, China). The levels of Aβ42 and Aβ40 in the brain hemispheres and femurs extracts were quantified using ELISA kits (Invitrogen, Camarillo, CA, USA).

Blood samples were obtained from posterior-orbital venous plexus, centrifuged at 5000 r/min for 15 min at 4°C, and then stored at −80°C for late use. The plasma was collected and stored in -80°C. Before analysis, the plasma samples were thawed to room temperature. The serum concentrations of TRACP 5b, IL-6 and TNF-α were assayed using ELISA kits (Jian Cheng, Nanjing, China). Assays were performed in accordance with the manufacturer’s recommendations.

### 2.4 Statistical analysis

All data were expressed as mean±SD. Statistical significance was set at *P* < 0.05, and determined by one-way analysis of variance and the SNK test with SPSS 19.0 (IBM, New York, USA). Graphs were drawn using GraphPad Prism (version 6.0 for Windows).

## Results

### 3.1 CUR prevented spatial memory deficit of APP/PS1 mutated transgenic mice

**Fig 1:**
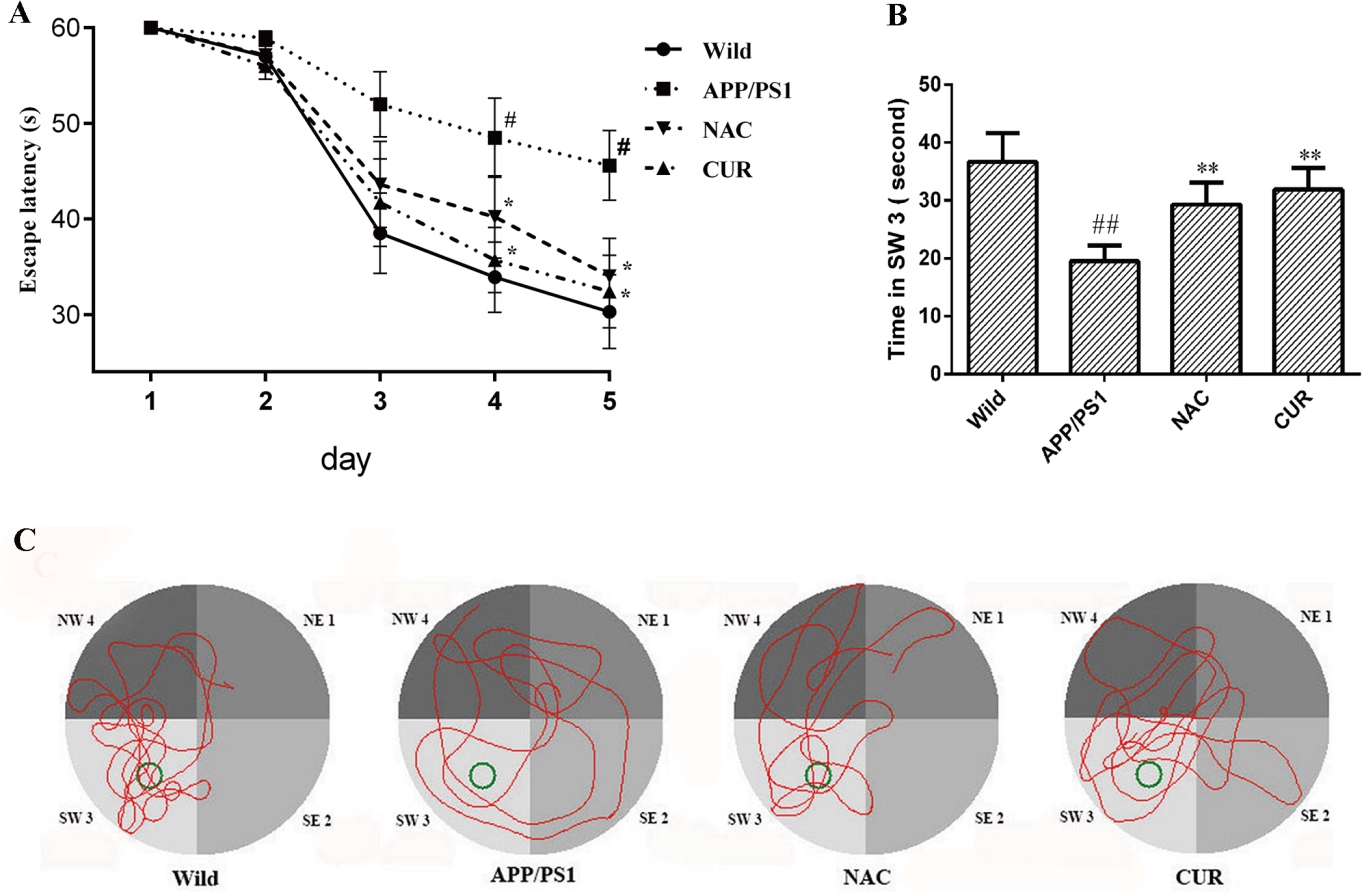
CUR prevented spatial memory deficit of APPPS1 mutated transgenic mice. Morris water maze was used to test the spatial learning and memory (n = 10). (A): CUR reduced the time to find the platform during the hidden sessions of the water-maze. (B): CUR treatment showed memeroy retention and spent most of their time in the target quadrant (SW 3) during the probe trial. (C): The route of the mice. Results are represented as the mean ± SD. #: *P*<0.05 compared to the wild mice group; ##: *P*<0.01 compared to the wild mice group *: *P*<0.05 compared to the APP/PS1 mutated transgenic mice group; **: *P*<0.01 compared to the APP/PS1 mutated transgenic mice group.

As shown in Fig 1A, during 5 day training period, the escape latency to find the platform decreased progressively. At the fourth week, the latency of APP/PS1 mice was longer than that of wild mice and this phenomenon was shortened by the treatment of CUR. In the probe trials (Fig 1B), the swimming time spent in SW 3was used to estimate retention performance. The swimming time of wild mice spent in SW 3 was 36.7 ±4.94 second. The APP/PS1 mice treated with CUR (31.9±3.70 second) swam in SW 3 longer than those in APP/PS1 group (19.5±2.71 second, *P*<0.01). Fig 1C shows the swim-paths of representative animals from four groups. The swimming tracks indicated that APP/PS1 mice searched for the platform in an inappropriate way and the mice in CUR and NAC treatment made it easy for the APP/PS1 mice to find the platform.

### 3.2 CUR reduced the levels of Aβ both in the brain and bone

**Fig 2:**
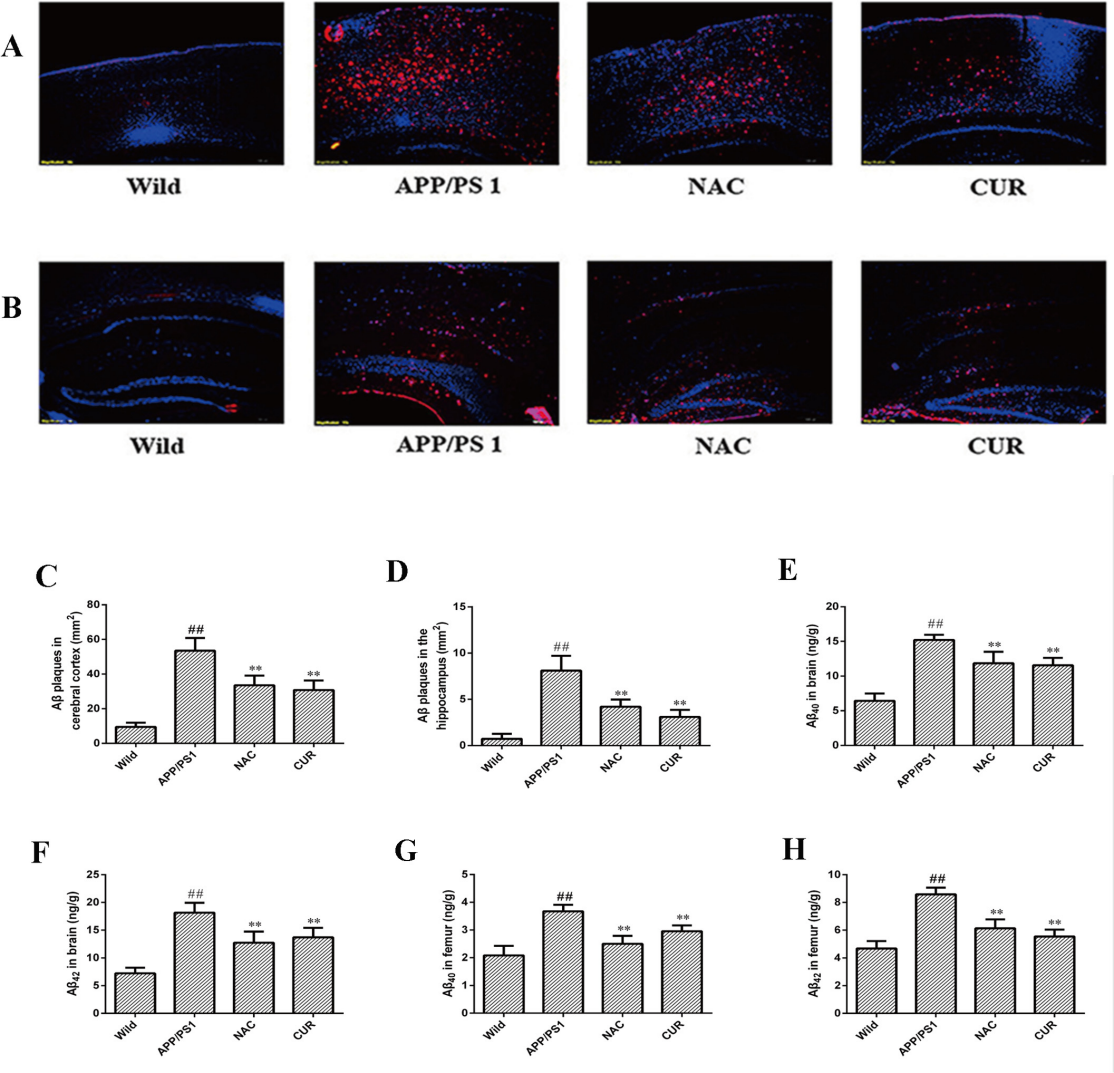
CUR reduced the levels of Aβ both in the brain and bone. Total Aβ plaques (red) were detected with antibodies in the cortex (A), and hippocampus (B). Cell nuclei were stained with DAPI (blue). Scale bars represented 100 μm. Number of total Aβ plaques plaques in the cortex (C) and hippocampus (D). CUR decreased the levels of Aβ40 (E) and Aβ42 (F) in brain, and decreased the levels of Aβ40 (G) and Aβ42 (H) in femur. Results are represented as the mean ± SD (n = 10). #: *P* < 0.05 compared to the wild mice group; ##: *P*<0.01 compared to the wild mice group *: *P*<0.05 compared to the APP/PS1 mutated transgenic mice group; **: *P*<0.01 compared to the APP/PS1 mutated transgenic mice group.

To determine the effect of CUR on the production and accumulation of Aβ, we observed the pathological changes of AD using immunohistochemistry and ELISA. CUR treatment markedly decreased the number of Aβ deposits (total β-amyloid peptide, including several isoforms of Aβ, such as Aβ40, Aβ42) both in the cortex (Fig 2A and 2C) and hippocampus (Fig 2B and 2D). As shown in Fig 2E and Fig 2F, Aβ40 and Aβ42 levels were significantly increased from 6.43±1.07 ng/g and 7.22±1.03 ng/g in the brain of wild mice to 15.21±0.76 ng/g (*P*<0.01) and 18.16±1.80 ng/g (*P*<0.01) in the brain of APP/PS1 mice. Brain Aβ42 and Aβ42 levels in the treatment of the CUR group decreased to 11.56±1.06 ng/g (*P*<0.01) and 13.72±1.72 ng/g (*P*<0.01) respectively. As well as in femur, Aβ40 and Aβ42 levels were significantly increased from 2.09±1.07 ng/g and 4.68±0.53 ng/g in wild mice to 3.67±0.24 ng/g (*P*<0.01) and 8.59±0.48 ng/g (*P*<0.01) in APP/PS1 mice. The Aβ42 and Aβ42 levels in the femurs of APP/PS1 mice treated with CUR decreased to 2.96±0.21 ng/g (*P*<0.01) and 5.55±0.49 ng/g (*P*<0.01).

### 3.3 The antioxidant effect of CUR in the brain and femur

**Fig 3:**
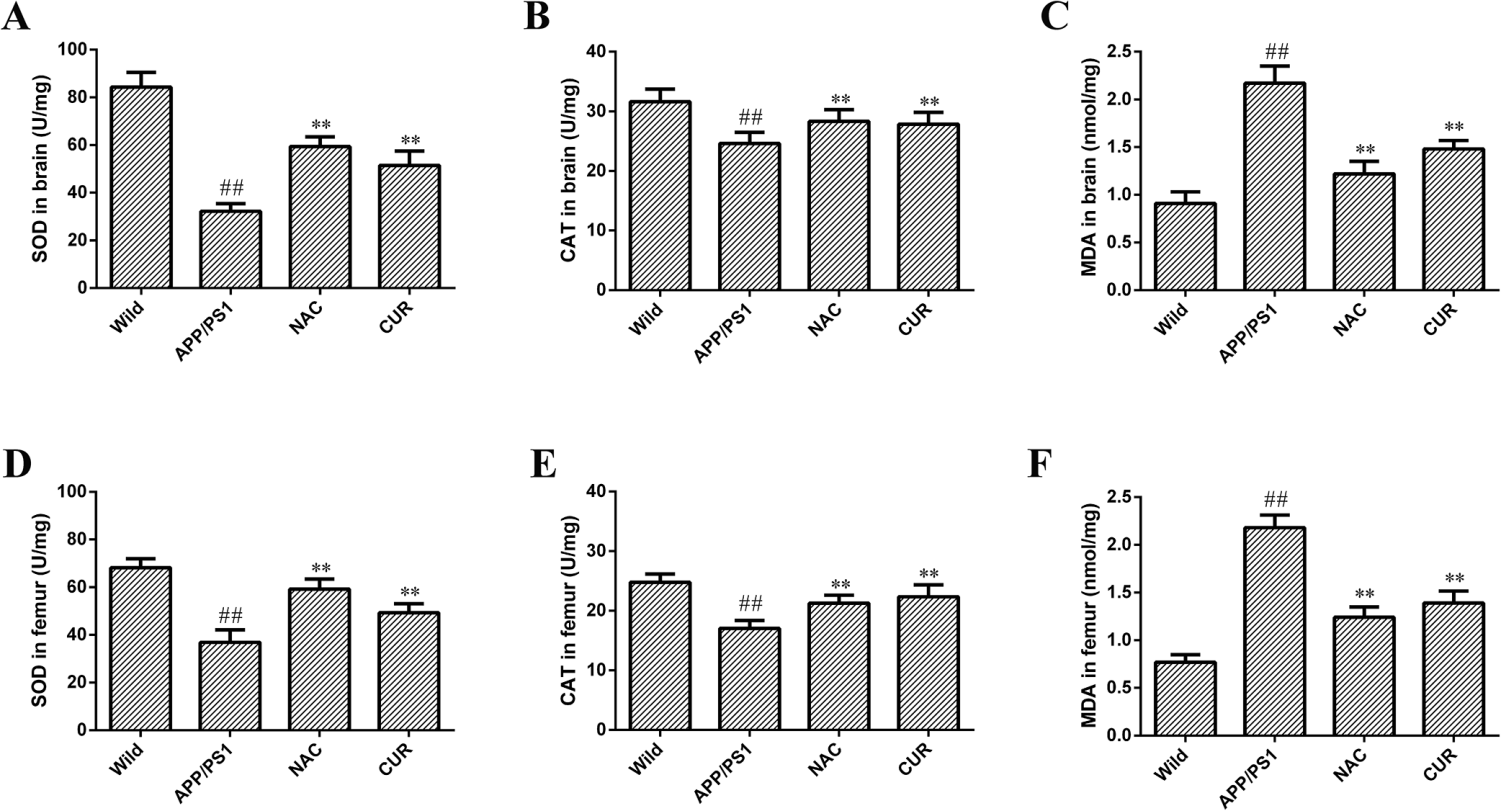
The antioxidant effect of CUR in brain and femur. The activities of antioxidant enzymes SOD (A), CAT (B) and the concentration of MDA (C) in brain, and SOD (D), CAT (E) and the concentration of MDA (F) in femur were assayed according to the instructions of the manufacturer. Results are represented as the mean ± SD (n = 10). #: *P*<0.05 compared to the wild mice group; ##: *P*<0.01 compared to the wild mice group *: *P*<0.05 compared to the APP/PS1 mutated transgenic mice group; **: *P*<0.01 compared to the APP/PS1 mutated transgenic mice group.

The SOD (Fig 3A) and CAT (Fig 3B) activities were respectively reduced from 84.30±6.17 U/mg protein and 31.59±3.11 U/mg protein in the brain of wild mice to 32.17±3.82 U/mg protein (*P*<0.01) and 24.61±1.87 U/mg protein (*P*<0.01) in the brain of APP/PS1 mice. The concentration of MDA (Fig 3C) was increased from 0.91±0.12 nmol/mg protein in the brain of wild mice to 2.17±0.18 nmol/mg protein in the brain of APP/PS1 mice (*P*<0.01). The activities of CAT and SOD were increased to 51.47±5.98 U/mg (*P*<0.01) and 27.84±2.01 U/mg (*P*<0.01) respectively, and the concentration of MDA was reduced to 1.48±0.09 nmol/mg (P<0.01). Additionally, in femurs of APP/PS1 mice, the activities of the antioxidative enzymes SOD (Fig 3D) and CAT (Fig 3E) were decreased significantly from 68.24±3.75 U/mg and 24.81±1.38 U/mg to 36.91±5.21 U/mg and 17.05±1.35 U/mg, and the levels of MDA (Fig 3F) were significantly elevated from 0.77±0.08 nmol/mg to 2.18±0.13 nmol/mg (*P*<0.01). Administration of CUR significantly restored SOD (49.29±3.71 U/mg) and CAT (22.34±2.01 U/mg) activities, and decreased MDA content (1.39±0.13 nmol/mg) in the femur (*P*<0.01). NAC supplementation preserved the SOD and CAT activities and decreased MDA content in the brain and femur.

### 3.4 CUR improved BMD and maintained bone structural properties of the femurs

**Fig 4:**
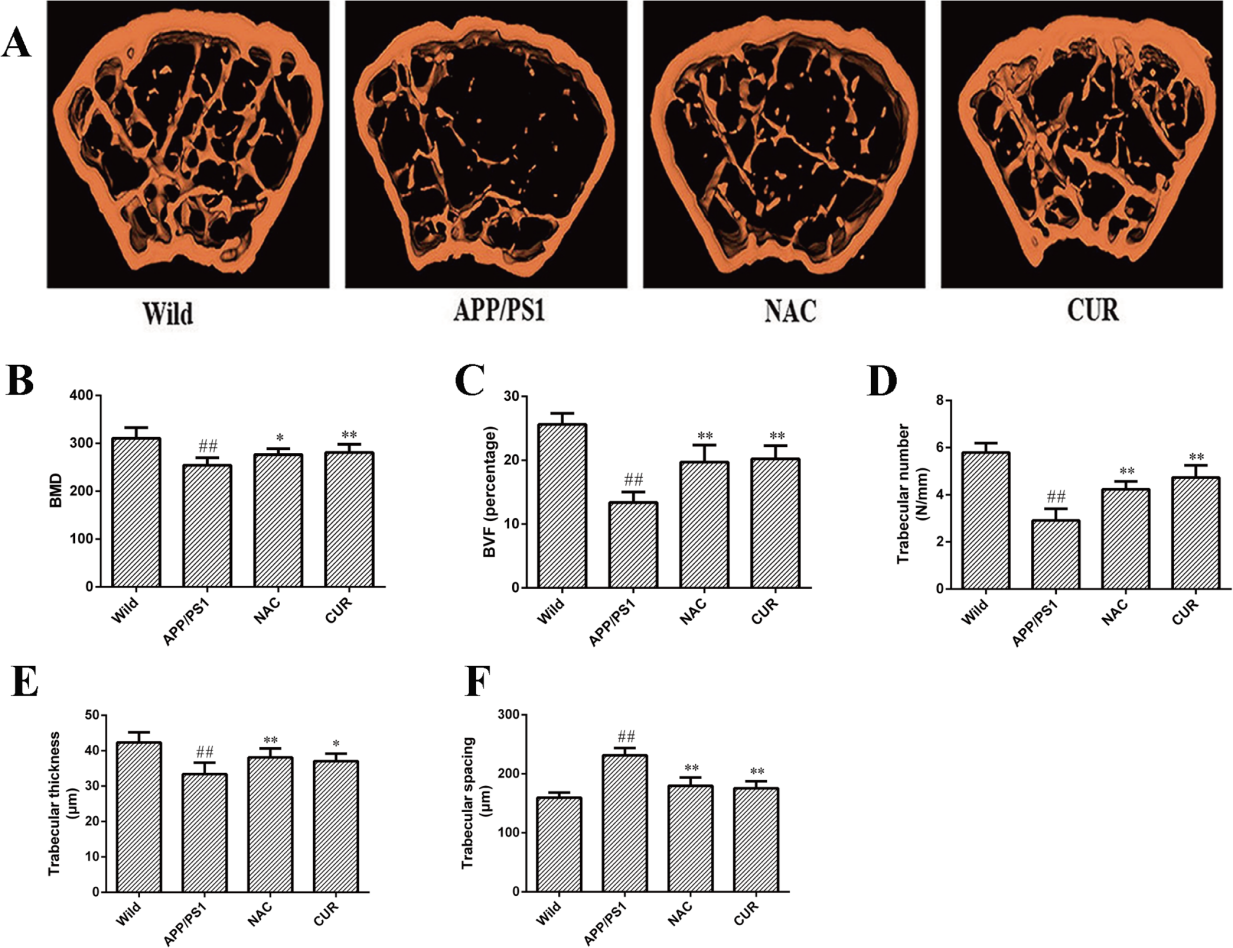
CUR improved BMD and maintained bone structural properties of the femur. Representative three-dimensional reconstructed images derived by micro–computed tomography (A). CUR treatment significantly improved Trabecular volumetric BMD (B) and increased BVF (C), trabecular number (C), trabecular thickness (E) and decreased trabecular separation (F). Results are represented as the mean ± SD (n = 10). #: *P*<0.05 compared to the wild mice group; ##: *P*<0.01 compared to the wild mice group *: *P*<0.05 compared to the APP/PS1 mutated transgenic mice group; **: *P*<0.01 compared to the APP/PS1 mutated transgenic mice group.

The micro-CT images of the distal femoral diaphysis showed BMD and bone structural properties of the trabecular bone in femurs in Fig 4A. Compared with the wild mice group, BMD (Fig 4B) of the femurs in APP/PS1 mutated transgenic mice decreased from 310.6±22.1 mg/mm^3^ to 254.3±15.9 mg/mm^3^ (*P*<0.01). As shown in Fig 4C–4F, the femurs in APP/PS1 mice decreased BVF (from 25.6±1.77 to 13.4±1.65 percentage), trabecular thickness (from 42.30±2.87 μm to 33.41±3.19 μm), and trabecular number (from 5.49±0.40 to 2.91±0.49), as well as a concomitant increase in trabecular spacing (from 159.70±8.69 μm to 231.40±12.54 μm). CUR treatment improved BMD (280.7±17.3 mg/mm^3^, *P*<0.01), decelerated the degeneration of trabecular bone, significantly increased BVF (20.21±2.05 percentage, *P*<0.01), trabecular thickness (37.08±2.09 μm, *P*<0.05), trabecular number(4.74±0.51, *P*<0.01), and decreased trabecular separation (175.62±11.67 μm, *P*<0.01) compared with model controls.

### 3.5 CUR improved the mechanical properties of the femurs

**Fig 5:**
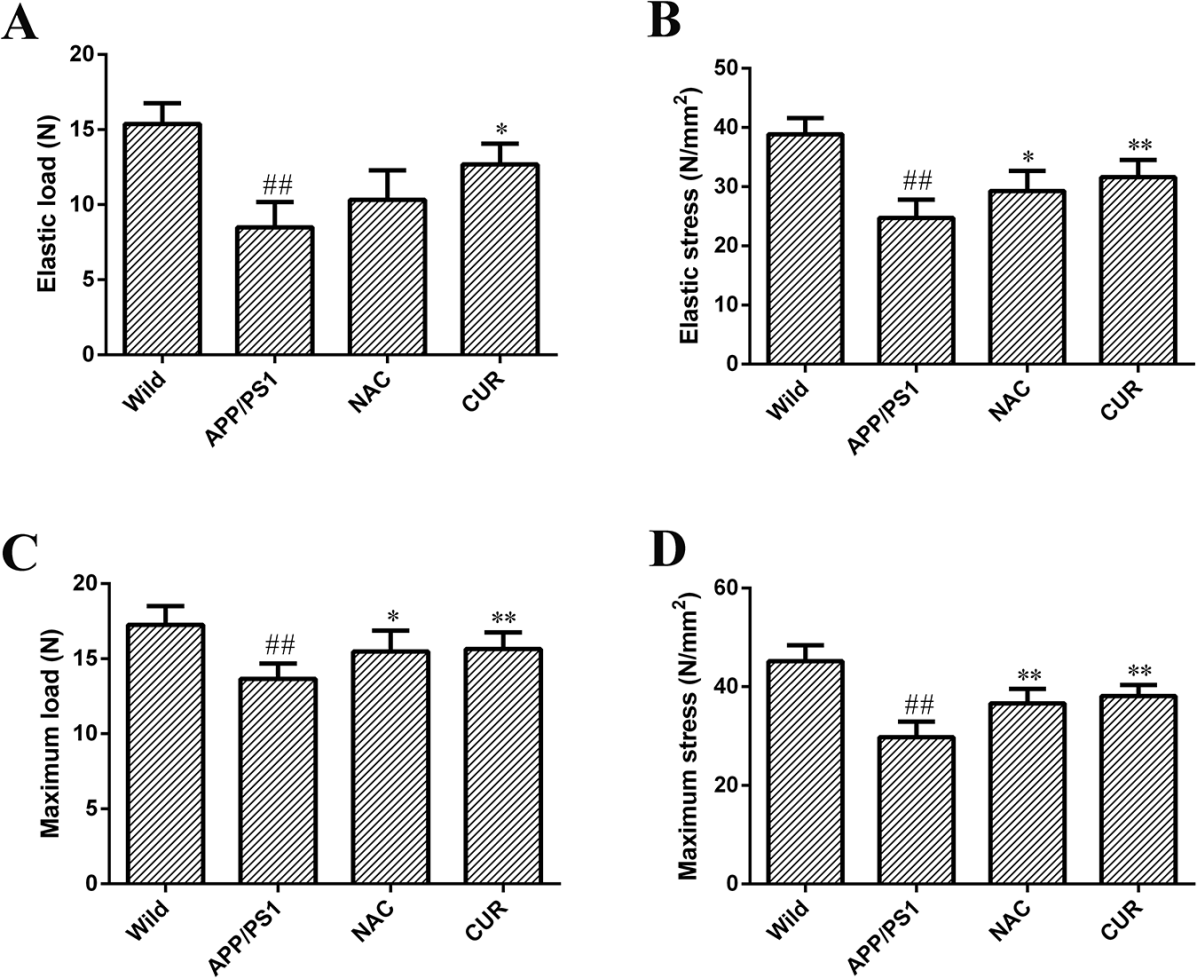
CUR improved mechanical properties of the femur. Elastic load (A), elastic stress (B), maximum load (C) and maximum stress (D) of the femurs were derived from the three-point bending test. Results are represented as the mean ± SD (n = 10). #: *P*<0.05 compared to the wild mice group; ##: *P*<0.01 compared to the wild mice group *: *P*<0.05 compared to the APP/PS1 mutated transgenic mice group; **: *P*<0.01 compared to the APP/PS1 mutated transgenic mice group.

A significant reduction in elastic load (Fig 5A), elastic stress (Fig 5B), maximum load (Fig 5C) and maximum stress (Fig 5D) of the femurs was observed between the APP/PS1 mice and the wild mice (*P*<0.01). The mechanical properties of the femurs, elastic load, elastic stress, maximum load and maximum stress were reduced from 15.37±1.38 N, 38.85±2.75 N/mm^2^, 17.25±1.25 N and 45.19±3.24 N/mm^2^ to 8.49±1.68 N, 24.71±3.11 N/mm^2^, 13.66±1.01 N and 29.78±3.12 N/mm^2^ respectively. The mice administered with 100 mg/kg/d CUR showed significant improvements in in elastic load (12.67±1.39 N, *P*<0.05), elastic stress (31.62±2.91 N/mm^2^, *P*<0.01), maximum load (15.64±1.11 N, *P*<0.01) and maximum stress (38.11±2.27 N/mm^2^, *P*<0.01) compared to model controls.

### 3.6 CUR reduced serum levels of TRACP 5b, IL-6 and TNF-α

**Fig 6:**
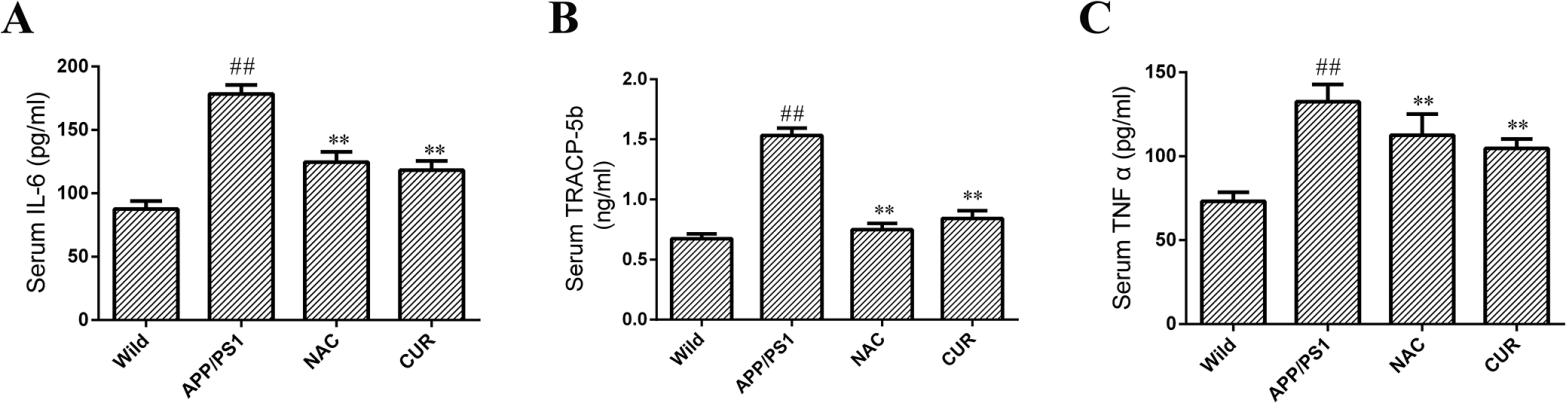
CUR reduced serum levels of IL-6, TRACP 5b and TNF-α. The serum concentrations of bone resorption markers including IL-6 (A), TRACP 5b (B) and TNF-α (C) were assayed using an ELISA kit. Results are represented as the mean ± SD (n = 10). #: *P* < 0.05 compared to the wild mice group; ##: *P*<0.01 compared to the wild mice group *: *P*<0.05 compared to the APP/PS1 mutated transgenic mice group; **: *P*<0.01 compared to the APP/PS1 mutated transgenic mice group.

The serum levels of cytokines IL-6 (Fig 6A), TRACP 5b (Fig 6B) and TNF-α (Fig 6C) were significantly elevated from 87.50±6.34 pg/ml, 0.674±0.040 ng/ml and 73.22±5.38 pg/ml to 178.41±7.21 pg/ml, 1.533±0.061 ng/ml and 132.51±10.25 pg/ml respectively in APP/PS1 mice compared with the wild group (*P*<0.01). CUR treatment reduced the levels of osteoclast activity markers TRACP-5b (0.842±0.066 ng/ml, *P*<0.01), and significantly decreased the levels of IL-6 (118.29±7.34 pg/ml, *P*<0.01) and TNF-α (104.70±5.67 pg/ml, *P*<0.01). These results suggested that CUR treatment was sufficient to decrease osteoclastogenesis induced by production and deposition of Aβ. As shown in S1 Fig, the serum levels of C-terminal cross-linking telopeptide oftype I collagen (CTx) and cathepsin K in APP/PS1 mice were significantly higher compared with the normal group. CUR and NAC reversed reduced the levels of osteoclast activity markers cathepsin K and CTx. CUR significantly reversed iron Aβ-induced down-regulated expression of osteocalcin (S2 Fig, *P*<0.01).

## Discussion

Alzheimer's disease (AD) and osteoporosis are two main multifactorial progressively degenerative diseases that predominantly affect the elderly. These two diseases share some common risk factors including old age, being female, smoking, excessive drinking, low estrogen, and vitamin D3 levels [27]. In agreement with the clinical and epidemiological evidence, oxidative damage and the dysfunction of the antioxidant system play an important role in the pathogenesis of osteoporosis and AD. In this study, we mimicked the prodromal stage of AD using APP/PS1 mutated transgenic mice, and proved the protective effect of CUR on the memory impairment and bone loss via anti-oxidative character.

Extracellular in the amyloid plaques and intracellular neurofibrillary tangles in the brain are primary characters for AD [28]. Amyloid plaques are more specific for AD, while neurofibrillary tangles occur in various neurodegenerative diseases [29]. It has been demonstrated that the mutations in the APP gene, presenilin (PS) 1, or PS 2 potentially lead to early-onset forms of AD [26]. In mice, the APP transgene combined with a PS1 transgene yielded Aβ plaques with earlier onset than the single transgenic sample [30]. Aβ directly or indirectly modulates mitochondrial function and induces oxidative stress which in turn enhances the Aβ synthesis and aggregation [26,31]. In our study, the administration of curculigoside significantly reduced the accumulation of Aβ and enhanced learning performance. Therefore, utilizing antioxidants may be a more successful strategy for the treatment of AD and osteoporosis.

In AD patients, SOD and CAT activities were found to be significantly lower in both the central nervous system and peripheral tissues[32]. The transgenic mice with overexpressing the APP mutant and a deficiency in Mn-SOD had elevated oxidative stress and significantly increased brain Aβ levels and Aβ plaque burden[33]. Conversely, the APP-overexpressing mutant mice with the overexpression of Mn-SOD, exhibited increased antioxidant defense capability in brains and reducing Aβ plaque burden [34]. In our study, APP/PS1 mice had significantly lower SOD, CAT activities, and higher levels of lipid peroxidation both in brain hemispheres and femurs. The anti-oxidative effects of CUR may be involved in the prevention of memory impairment and bone loss.

In bone tissue, oxidative stress associated with aging and estrogen deficiency may be a pivotal pathogenetic mechanism of bone loss [35]. Women with postmenopausal osteoporosis have significantly lower SOD, GPX, CAT activity and higher levels of lipid peroxidation end-product MDA, and the antioxidant enzymes levels are significantly associated with BMD values of the femoral neck, lumbar spine, and total hip [36,37,38]**。**. In bone marrow stromal cell and calvarial osteoblast, oxidative stress inhibits osteoblastic differentiation mainly through the activation of extracellular signal-regulated kinase (ERK) and ERK-dependent NF-κB signaling pathways [39].

Osteoblasts can also produce receptor activator for nuclear factor-κ B Ligand (RANKL) and osteoprotegerin (OPG) to modulate osteoclast differentiation and bone resorption. In our previous study, CUR significantly down-regulated the increased level of RANKL in H_2_O_2_-stimulated osteoblast, and exhibited potential effects on restraining bone absorption [15].

In addition, Aβ also plays a crucial role in the demineralization process of bone tissues of older people and women with menopause. The mRNA and protein expression levels of Aβ42 and APP were elevated remarkably in the osteoporotic bone tissues both from human and ovariectomized rats [7]. In our study, the levels of Aβ in the femurs of APP/PS1 mutated transgenic mice were significantly elevated. Aβ40 and Aβ42 levels were significantly decreased in the CUR treated group. In addition, the elevated level of Aβ induced higher levels of bone resorption marker cathepsin K and CTx, and lower level of biochemical marker of bone formation (osteocalcin).

In APP/PS1 mice, the elevation of TNF-α and IL-6 is closely related to bone loss [40]. The concentrations in the serum of IL-6 and TNF-α were associated with increased ROS and bone resorption [41]. In our research, the serum levels of the osteoclast activity markers (TRACP 5b), IL-6 and TNF-α were significantly higher in APP/PS1 mice than in the wild mice. IL-6 and TNF-α not only directly stimulate osteoclastogenesis and bone resorption but also stimulate RANKL production in osteoblastic cells in a synergistic fashion [42]. CUR treatment reduced the levels of osteoclast activity markers TRACP-5b, and significantly decreased the levels of IL-6 and TNF-α. These results suggested that CUR treatment was sufficient to decrease osteoclastogenesis induced by production and deposition of Aβ.

In conclusion, the administration of CUR can significantly enhance learning performance and ameliorate bone loss in APP/PS1 mutated transgenic mice, and the mechanism may be related to its antioxidant effect.

## Supporting Information

S1 Fig CUR reduced serum levels of cathepsin K and CTx. The serum concentrations of cathepsin K and C-terminal cross-linking telopeptide oftype I collagen (CTx) were assayed using an ELISA kit. Results are represented as the mean ± SD (n = 10). #: *P* < 0.05 compared to the wild mice group; ##: *P*<0.01 compared to the wild mice group *: *P*<0.05 compared to the APP/PS1 mutated transgenic mice group; **: *P*<0.01 compared to the APP/PS1 mutated transgenic mice group.
(TIF)

S2 Fig CUR reduced serum level of osteocalcin. The serum concentration of osteocalcin was assayed using an ELISA kit. Results are represented as the mean ± SD (n = 10). #: P < 0.05 compared to the wild mice group; ##: P<0.01 compared to the wild mice group *: P<0.05 compared to the APP/PS1 mutated transgenic mice group; **: P<0.01 compared to the APP/PS1 mutated transgenic mice group.
(TIF)
