# Supplementary figures and images for: Effects of Curculigoside on Memory Impairment and Bone Loss via Anti-Oxidative Character in APP/PS1 Mutated Transgenic Mice
Source: PLoS One. 2015 Jul 17;10(7):e0133289. doi: 10.1371/journal.pone.0133289 (PMC4505858; doi:10.1371/journal.pone.0133289)

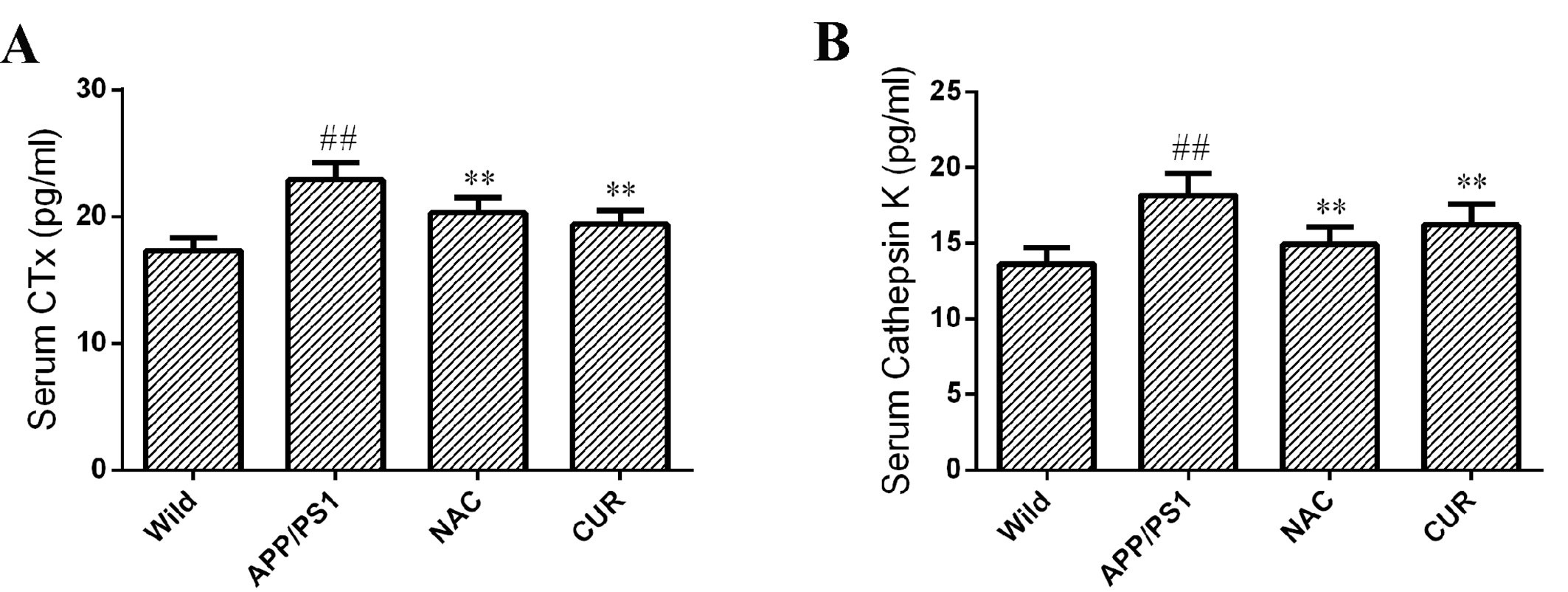

Supplement: S1 Fig — The serum concentrations of cathepsin K and C-terminal cross-linking telopeptide oftype I collagen (CTx) were assayed using an ELISA kit. Results are represented as the mean ± SD (n = 10). #: P < 0.05 compared to the wild mice group; ##: P<0.01 compared to the wild mice group *: P<0.05 compared to the APP/PS1 mutated transgenic mice group; **: P<0.01 compared to the APP/PS1 mutated transgenic mice group. (TIF) [file pone.0133289.s001.tif]
